# Insights Into the Current and Future State of AI Adoption Within Health Systems in Southeast Asia: Cross-Sectional Qualitative Study

**DOI:** 10.2196/71591

**Published:** 2025-06-16

**Authors:** Mochammad Fadjar Wibowo, Alexandra Pyle, Emma Lim, Joshua W Ohde, Nan Liu, Jonas Karlström

**Affiliations:** 1 SingHealth Duke-NUS Global Health Institute Duke-NUS Medical School Singapore Singapore; 2 Department of Future Health System Singapore General Hospital Singapore Singapore; 3 Global Public Health, Global Studies University of Virginia Charlottesville, VA United States; 4 Center for Digital Health Mayo Clinic Rochester, MN United States; 5 Centre for Quantitative Medicine Duke-NUS Medical School Singapore Singapore

**Keywords:** artificial intelligence, AI, digital transformation, digital health, technology adoption, health system integration, accessibility, governance, Southeast Asia

## Abstract

**Background:**

Artificial intelligence (AI) holds potential to enhance health systems worldwide. However, its implementation in health systems in Southeast Asia (SEA)—a region of diverse geopolitical and socioeconomic development—has been understudied.

**Objective:**

This study aims to gain insights into the current state and future prospects of AI technology from participants most directly involved in its adoption across health systems in SEA whose perspectives have received limited attention in research to date.

**Methods:**

We used a cross-sectional qualitative research design. Data were collected through 31 semistructured interviews with participants working in or significantly involved with the implementation of AI-enabled technologies within health systems across 7 SEA countries: Brunei Darussalam, Indonesia, Myanmar, Singapore, Thailand, Vietnam, and the Philippines. The participants represented the public, private, and nonprofit sectors. They included innovators, health care professionals using AI, professionals from nongovernmental and multilateral organizations, corporate professionals, academics, policy makers, regulators, and investors. All interviews were audio recorded and transcribed verbatim. The collected data were then analyzed using thematic analysis methodology to identify key themes.

**Results:**

Of the 31 participants, 8 (26%) were from lower–middle-income countries, 16 (52%) from upper–middle-income countries, and 7 (22%) from high-income countries. Through thematic analysis, five major categories emerged: (1) AI technology acceptance, (2) disparities in digital transformation, (3) technology governance, (4) data governance, and (5) AI for health system transformation. Participants discussed the promise of AI technology for adoption and integration in the health sector. In lower–middle-income and upper–middle-income countries, disparities in digital transformation—such as infrastructure barriers, market access concerns, and limited investment—were viewed as critical impediments. Across all country income levels, technology and data governance were considered essential for the ethical integration of AI into health care systems. AI is perceived to have the potential to transform health systems, including population health management, service accessibility, operations management, health systems financing and health care payment, and personalized medicine.

**Conclusions:**

Our study provides novel perspectives and valuable insights into the current state and future prospects of AI adoption across health systems in SEA. By capturing the experiences and opinions of a broad range of professionals involved in health care and AI, this research provides a nuanced understanding of the opportunities and hurdles associated with health AI in the region. For the full potential of AI-enabled technologies to be successfully implemented and ultimately contribute to the transformation of health systems in the region, foundational investments are needed in digital infrastructure, technology governance, and data governance. These fundamental pillars are crucial for fostering an environment in which AI can be effectively and ethically leveraged to improve health outcomes and strengthen health care systems throughout SEA.

## Introduction

### Background

Various digital health technologies have revolutionized health care by creating a more integrated, efficient, and decentralized approach to delivering health care services [1]. Among these, recent developments in artificial intelligence (AI) have emerged as a transformative force in health systems, contributing to improved diagnostics, optimized clinical protocols, enhanced health care management, and strengthened supply chain logistics [2]. During the COVID-19 pandemic, the application of AI technologies accelerated digital health transformation globally, with many countries rapidly adopting telemedicine and AI-assisted surveillance, including serological surveillance tools to understand population immunity, to manage the crisis [3]. These experiences may have shifted stakeholder attitudes toward AI from skepticism to cautious optimism [4]. In high-income settings, health-related AI is positioned to transform the delivery of care, particularly in specialized fields such as ophthalmology [5], radiology, cardiology, and pathology [6].

In Southeast Asia (SEA)—a region with heterogeneity in geopolitics and socioeconomic development stages [7]—the levels of national health AI adoption and integration remain uneven and understudied [8]. According to the World Health Organization, successful AI integration must be built upon a comprehensive strategy that addresses the digital divide with accessible, scalable, and person-centric health solutions tailored to local contexts and regional priorities [9]. Moreover, investing significant resources into developing AI without first building the necessary digital and knowledge infrastructure foundation needed for AI is unethical [10].

Aligning AI adoption with SEA’s health care priorities, such as universal health coverage (UHC), primary health care (PHC) improvements, and disease surveillance, is crucial. The World Health Organization’s regional strategy for UHC in SEA underscores the importance of strengthening PHC as a pathway to achieving UHC and improving health outcomes [11]. AI can enhance PHC by improving diagnostics, facilitating remote consultations, and optimizing resource allocation, thereby contributing to more efficient and equitable health care delivery. In addition, AI-driven disease surveillance systems can enable timely detection of and response to outbreaks, bolstering regional health security [11].

Understanding the perspectives of multiple stakeholders through participants who are significantly involved in introducing AI technologies in SEA’s health care sector is essential. Globally, studies providing new insights into the current state, criteria, challenges, and outlook for implementing AI technologies in health care from the perspectives of experts and practitioners [12] primarily originate from western Europe [13] and North America [14]. In Asia, the barriers and facilitators of health AI technology development and implementation have been studied mainly in China and India, while in SEA, views about health AI adoption are limited and primarily from Singapore, Thailand, and Vietnam [15]. This narrow geographic coverage limits our understanding of how AI technologies are perceived, adopted, or resisted in diverse SEA contexts, where differences in health systems governance, financing, and workforce readiness could yield markedly different outcomes.

### Objectives

Given this gap, there is an urgent need for regionally grounded studies that reflect the voices of participants who are most directly involved in the design, implementation, and regulation of AI in SEA health systems. Considering the region’s diversity in languages, cultures, and economic development [16], the current limited empirical research on AI adoption and integration across SEA highlights a need to expand knowledge in this area. Therefore, we aim to understand participants’ perspectives regarding the integration of inclusive health AI into health systems that address diverse and contextual needs in SEA.

This study explores the current and future state of health AI adoption and integration across SEA. Drawing on in-depth insights from participants involved in AI implementation across public, private, and nonprofit sectors in 7 SEA countries, we aim to identify a broad range of perceived barriers and enablers as well as examine the contextual factors that influence AI readiness and integration within diverse national health systems.

## Methods

### Study Design

We used a standard qualitative research approach [17]. Qualitative methods were selected over quantitative surveys or mixed methods because this approach is most appropriate for elucidating the in-depth and real-life experiences, opinions, and attitudes of participants, which supports efforts to address the aforementioned aim of this research. More specifically, it is an apt method to discover how and why localized contextual factors, such as sociocultural or political situations, impact the current and future state of health systems [18]. This study is reported in accordance with the COREQ (Consolidated Criteria for Reporting Qualitative Research) checklist [19] (Multimedia Appendix 1).

### Ethical Considerations

This study was reviewed and approved by the National University of Singapore Institutional Review Board (NUS-IRB-2023-562) and was deemed to involve less than minimal risk to participants, thus exempt from further review. Verbal consent was obtained from all participants before the commencement of the interviews and audio recordings. All data collected were anonymized during transcription, with any identifying information removed to protect participant privacy. Access to the anonymized data was restricted to the research team and securely stored. Participants did not receive any monetary or material compensation for their participation. Their involvement was entirely voluntary. Verbal consent was obtained from all participants before the start of the interviews and audio recordings.

### Study Population and Sampling

Purposive sampling was used to target experts involved in AI adoption across health care services and systems in SEA [20]. Participants were recruited through the authors’ networks and were contacted via email and LinkedIn (LinkedIn Corporation). Snowball sampling further expanded the participant pool. Participants were eligible for inclusion if they held mid- to senior-level roles; had ≥10 years’ experience in health care technology across private, public, and nonprofit sectors in SEA; and were involved in health AI at the time the study was conducted. To ensure diverse perspectives, we sought representation from 8 distinct categories: innovators, health care professionals using AI (private and public), professionals from nongovernmental and multilateral organizations, private sector AI health professionals, academics, policy makers and government representatives, regulators, and investors. To facilitate this, when identifiable, we strategically connected with key organizations and networks within each country; for instance, in Vietnam, we reached out to the Vietnam Association of Medical & Biological for Research and Application, which is based in Hanoi. In Indonesia, we engaged with a contact from a health information system technical working group based in Jakarta. In Thailand, we connected with the Thai Health Information Standards Development Center in Bangkok.

### Data Collection and Analysis

Individual semistructured interviews were held either in person or via Zoom (Zoom Video Communications, Inc) between June 16 and July 29, 2023. Each interview lasted 45 to 60 minutes. We developed an interview guide (Multimedia Appendix 2) to facilitate the discussion. The interview questions explored specific areas, including the use of AI to improve health outcomes, protect the health of populations, and strengthen health care systems; AI challenges in health care; concerns regarding AI development and use in participants’ occupation or professional setting; future capabilities of, and opportunities for, health care AI; and proactive measures to maximize the benefits of AI participant demographics, including sex, profession, employment sector, and country. All interviews were audio recorded and transcribed verbatim using Otter.ai (Otter.ai, Inc) [21].

Data were analyzed using thematic analysis in Microsoft Excel. The analysis was conducted in 6 phases (Multimedia Appendix 3 [17,21]). To minimize potential researcher bias throughout the analysis process, collaborative coding was performed while maintaining an audit trail of analytical decisions. MFW, EL, and AP independently coded a subset of transcripts, followed by regular meetings to compare initial codes, resolve discrepancies, and agree on a shared coding framework. Codes were accordingly clustered into potential main themes based on conceptual similarity. Subthemes were then created to capture nuanced dimensions within broader themes. All themes were reviewed and refined through ongoing comparison with the raw data and an assessment of their internal coherence and external distinction. This iterative process ensured that the themes accurately reflected the dataset and our research aims. Thematic saturation was reached when no new themes emerged from the data, and the representativeness and consistency of topics were achieved. The sample size was determined when saturation was reached because additional interviews would no longer contribute new information. A final thematic table was agreed upon by the research team members, who collectively validated its relevance and representation.

### Reflexivity

Most of the interviews (23/31, 74%) were conducted in English. MFW interviewed 8 (67%) of the 12 Indonesian participants in Bahasa Indonesia to cater to the interviewees’ native language. To minimize interpretive challenges, MFW also translated these transcripts from Bahasa Indonesia into English. This was deemed appropriate because MFW is bilingual in both languages, aware of cultural nuances, and well versed in the study context. Although the authors did their utmost to address reflexivity throughout the analysis process, they are cognizant that some language choices in the translation process may have influenced the coding and theme development.

## Results

### Participants

We invited 131 potential participants, of whom 36 (27%) provided a response. Of these 36 respondents, 31 (86%) completed an interview. The interviewees represented eight professions: (1) innovators, (2) health care professionals using AI, (3) professionals from nongovernmental and multilateral organizations, (4) corporate professionals, (5) academics, (6) policy makers, (7) regulators, and (8) investors. Of the 31 participants, 8 (26%) were from lower–middle-income countries (LMICs), 16 (52%) from upper–middle-income countries (UMICs), and 7 (23%) from high-income countries (HICs)—as defined by the World Bank [22]. Participants from Laos and Malaysia were also recruited, but they were unable to complete an interview within the timeline of the study’s data collection period. We could not recruit participants from Cambodia because we were unable to establish network contacts within the country. Overall, 84% (26/31) of the participants were male. Table 1 reports the participant characteristics.

**Table 1:** Participant characteristics (n=31).

Characteristics		Participants, n (%)
**Sex**		
	Male	26 (84)
	Female	5 (16)
**Profession**		
	Innovators	5 (16)
	Health care professionals using AI^a^	3 (10)
	Professionals from nongovernmental and multilateral organizations	6 (19)
	Corporate professionals	4 (13)
	Academics	8 (26)
	Policy makers	2 (6)
	Regulators	1 (3)
	Investors	2 (6)
**Sector**		
	Public	10 (32)
	Private	14 (41)
	Nonprofit	7 (23)
**Country (World Bank classification [22])**		
	Brunei Darussalam (HIC^b^)	1 (3)
	Indonesia (UMIC^c^)	12 (39)
	Myanmar (LMIC^d^)	3 (10)
	Singapore (HIC)	7 (23)
	Thailand (UMIC)	4 (13)
	Vietnam (LMIC)	3 (10)
	Philippines (LMIC)	1 (3)

^a^AI: artificial intelligence.

^b^HIC: high-income country.

^c^UMIC: upper–middle-income country.

^d^LMIC*:* lower–middle-income country.

Five main themes emerged: AI technology acceptance, disparities in digital transformation, technology governance, data governance, and AI for health system transformation. Descriptions of the main themes and subthemes with exemplar quotes are presented in the following subsections.

### AI Technology Acceptance

The majority of the participants recognized that to adopt AI within a health care setting, acceptance toward AI technology as a whole is needed. While participants from HICs tended to emphasize the sophistication of technology integration, those from LMICs focused on the foundational need for basic acceptance before advancing further.

#### Perceived Risks and Resistance

As indicated by participants P18 and P20 (Table 2), factors hindering AI technology acceptance were predominantly related to the perception of risks and resistance. On one hand, concerns were raised about potential overreliance or dependence on AI. On the other hand, a lack of trust in AI systems was noted by some health care professionals using AI, reinforcing the essential role of human oversight in AI applications. A small number of participants raised concerns about the accuracy of AI in clinical decision support and explicitly referred to safety implications for patient welfare. Resistance was noticeable in remote areas. Some participants asserted that physicians are anxious about the prospect of AI replacing them, while others highlighted the challenges that face physicians who do not adopt AI technologies. This situation seemed more pronounced among participants from LMICs, where resource constraints and infrastructural deficits compounded these fears, whereas participants from UMICs and HICs were more likely to engage in discussions on balancing innovation with risk management.

**Table 2:** Artificial intelligence (AI) technology acceptance.

Main theme and subthemes		Example quotes
**AI technology acceptance**		
	Perceived risks and resistance	“In the past, we developed a simple rule-based system to determine diagnoses based on input data. This system, intended for remote areas with a shortage of health workers, met with resistance, especially from clinicians who felt controlled by the system’s dictations. This reflects the prevalent hesitation towards embracing this technology, hinting at potential resistance to AI implementation in health care.” [P18, professional from NGO^a^ and multilateral organization, Myanmar] “Although there is legislation in place that requires AI used in hospitals to be certified, sometimes certain setups acquire data from the internet and materials without obtaining proper certification before directly implementing them in hospitals. This can potentially harm patients, and there have been reported cases of such incidents.” [P20, academic, Thailand]
	Addressing human factors	“They [patients] still want like some form of human connection, touch...they would love to see the person as well because there are much more data that is required because the AI can only provide certain amount of information, even the input data, right?” [P31, academic, Thailand] “Ease of use is another factor. If a hospital only maintains paper-based records and you introduce an AI system, that system must be intuitive. Through our work on a digital health road map with the World Heart Federation, we found that if an application is difficult to use, it is less likely to be adopted, particularly if the benefits are not immediately apparent. Therefore, the design must be user-friendly and the system’s effectiveness should be clearly communicated to its intended users, whether they’re patients, physicians, or nurses. These barriers exist at all levels.” [P11, academic, Singapore]

^a^NGO: nongovernmental organization.

#### Addressing Human Factors

Ensuring optimal, user-centered human factors in AI integration within health care was viewed as essential for AI acceptance. Participants anticipated changes in patient-physician interactions and observed that some patients prefer human interaction over interacting with a bot, which highlighted AI’s limited human touch. It was stated that this is further complicated because some patients find advanced diagnostic tools provided by AI to be unintuitive, emphasizing the significance of designing AI systems that are user-friendly and accessible to all patients. Table 2 offers examples of supporting quotes from participants P31 and P11 that reflect this subtheme. Across different country income settings, the nature of the challenge varied: in LMICs, the focus was on overcoming basic usability hurdles, whereas in HICs, efforts centered on enhancing the digital experience without compromising the personal element of care.

### Disparities in Digital Transformation

Participants highlighted disparities in digital transformation as a fundamental problem, exposing various systemic barriers that complicate the implementation and scalability of AI technologies. Notably, the disparities in digital transformation were strongly influenced by economic context: LMICs typically confronted more basic infrastructural and regulatory challenges, while HICs grappled with optimizing already advanced systems for efficiency and integration.

#### Infrastructure as a Barrier to AI Adoption

As reflected by the example quotes from participants P4 and P13 (Table 3), the interviewees generally expressed that factors contributing to the limited digital transformation within their home country was the result of inadequate basic infrastructure (eg, unreliable internet and electricity). This view was particularly common among participants from LMICs. Furthermore, concerns about the varying quality of and access to health care facilities and data were raised, demonstrating the uneven development of infrastructure across the region. Participant P24 (Table 3) specifically expressed that in Myanmar, the political situation was considered a major disruptor to adequate infrastructure development and health care delivery. As such, resolving political instability was noted as a prerequisite for digitizing health care and subsequently adopting health AI. By contrast, while participants from UMICs and HICs also acknowledged infrastructural challenges, the issues they highlighted were more closely tied to system interoperability and the integration of cutting-edge technologies rather than the absence of basic services.

**Table 3:** Disparities in digital transformation.

Main theme and subthemes		Example quotes
**Disparities in digital transformation**		
	Infrastructure as a barrier to AI^a^ adoption	“I can see that Vietnam has a long way to go in terms of AI. Working in the field, we realize that the EMR [electronic medical record], the HISS [hospital information support system], cardiology information system, all those systems are nonexistent for now, or very low, maybe 5% or not even 10% for sure...In Vietnam, where my expertise lies, the main difficulty is the level of digitalization. To be smart, you need to digitize the system, but the degree of digitalization in Vietnam is very low. Most things are still done manually, and digitizing health care operations is costly.” [P4, investor, Vietnam] “To promote digital technology adoption, we need to address the accessibility to the internet, electricity, and infrastructure for the general population. These are fundamental issues that need to be resolved before expecting widespread adoption. People will naturally raise concerns about these root causes and political aspects.” [P13, professional from NGO^b^ and multilateral organization, Myanmar] “I think one of the first challenges before implementing AI is the political situation. Currently, the focus is mainly on political institutions, and there is not much focus on public health or health care. For countries like Myanmar, peace and stability are crucial before implementing AI in health care.” [P24, academic, Myanmar]
	Market access concerns	“Entering the government market is unpredictable, which is widely known.” [P9, regulator, Indonesia] “And for the hospital to buy a server, it’s not so easy in Vietnam because of, you know, the administrative constraints and many things.” [P19, academic, Vietnam]
	Limited investment	“Financing is a significant obstacle as implementing AI systems in health care requires substantial capital investment.” [P19, academic, Vietnam] “We are working to bring AI into APAC [Asia-Pacific region] ourselves, but there’s an issue with pricing. The cost for each case is quite high.” [P4, investor, Vietnam]

^a^AI: artificial intelligence.

^b^NGO: nongovernmental organization.

#### Market Access Concerns

Participants from LMICs cited challenges with the fast and continuous introduction of AI to the market as barriers to potential AI adoption. Interviewees from Indonesia, Myanmar, and Thailand specified the unpredictability of the public sector market and limited government influence over the private sector as major obstacles. This interpretation is supported by an example quote from participant P9 (Table 3). In addition, it was noted that the competitive nature of the telemedicine sector in Indonesia as well as the administrative constraints in Vietnam (refer to the quote from participant P19 [Table 3]) further complicate the market access landscape. Conversely, participants from UMICs and HICs often reported more stable market conditions, with the discussion shifting toward refining regulatory frameworks and ensuring sustainable innovation rather than merely securing market entry.

#### Limited Investment

The example quotes from participants P19 and P4 (Table 3) illustrate how financial barriers, such as restrictive funding regulations, the high cost of innovation, and scant public sector investment, severely hinder AI development and implementation. Participants also acknowledged that the reliance of underdeveloped economies on international aid, rather than investing in domestic initiatives, poses a challenge to innovation because the aid may cease at any time. This financial disparity underscored how economic development influenced AI adoption. While LMICs faced a critical need for foundational investment, HICs may have leveraged more robust financial ecosystems to foster innovation despite their own fiscal constraints.

### Technology Governance

Technology governance emerged as an important enabler for the ethical integration of health AI.

#### Need for Ethical and Regulatory Frameworks

Many participants emphasized the need for a government road map to guide AI implementation, underscoring the importance of establishing ethical risk management and regulations to ensure AI transparency. The example quotes from participants P22 and P8 (Table 4) support this interpretation. Participants also expressed that implementing liability protections for physicians is essential to foster trust in the use of AI for clinical decision-making. Table 4 displays a key statement from participant P3 that aligns with this viewpoint. In HICs, such frameworks were often more advanced, reflecting a higher level of systemic trust and regulatory maturity, while in LMICs, the call for such frameworks was part of a broader need for digital infrastructure and governance reforms.

**Table 4:** Technology governance.

Main theme and subthemes		Example quotes
**Technology governance**		
	Need for ethical and regulatory frameworks	“Top of mind, I would say, is the lack of guidelines. In any innovative field, the pioneers often tread in gray areas. Meaning, it’s unclear to regulators and government officials what can and cannot be done. This lack of clarity presents a significant challenge.” [P22, innovator, Philippines] “There are no rules regarding what diseases can be diagnosed by teleconsultation. Not to mention discussing how AI [artificial intelligence] can help in the treatment of diseases according to existing regulations.” [P8, health care professional, Indonesia] “Is there legal protection for doctors if something goes wrong? This is a big concern for doctors in Indonesia.” [P3, innovator, Indonesia]
	Capacity building for digital health care transformation	“The challenge lies in bridging the gap between those who are tech savvy but lack health knowledge and those who understand health but struggle with technology...Increasing data literacy and promoting the potential of data analytics at the city, district, and provincial levels can significantly drive progress.” [P5, academic, Indonesia] “People need a certain level of literacy to understand the benefits of this system and how they can use it to improve their health.” [P6, health care professional, Indonesia]
	Need for multistakeholder and interagency collaboration	“For AI to positively impact health care in Indonesia, it is essential to foster a collaborative approach that brings together domain experts in health care and technology professionals.” [P12, corporate professional, Indonesia] “Collaboration between academia, industry, and health care professionals will be essential to drive innovation, validate AI algorithms, and ensure their effective integration into the health care system. Overall, a comprehensive and collaborative approach involving the government, private sector, and health care stakeholders is necessary to achieve the full potential of AI in health care in Thailand.” [P20, academic, Thailand] “My worry is, are we to accept an imperfect AI experience at the expense of the overall health care experience? This thought keeps me awake at night, pondering how we can address this issue. One solution might involve a coalition or fellowship. If certain parties can contribute baseline training models or customized models to a certain level, then other members could continue with the more intimate localization efforts. This way, we’re working together towards a common goal rather than competing in separate races. Public health systems could take the lead on this, representing the greater good. While the private sector primarily focuses on profitability, health care also serves a social purpose. The government or public health sector should take on certain responsibilities or challenges, such as the provision of basic health care services. If digital health, data availability, or AI capability become part of the basic provision of health, we’ll need to account for that as well.” [P21, investor, Singapore]
	Need for contextualized research and development and innovation	“I work extensively in implementation science, so I might be biased, but I believe that if we conduct local effectiveness and acceptability studies, acceptance will be higher.” [P22, innovator, Philippines] “The second problem is the lack of accuracy when applied to our Thai patients. Although the solutions look very promising in terms of military applications and research, with high accuracy when using their own data, the performance doesn’t translate well to the field setting with Thai citizens. This is a significant deviation from the paper proposals. This is why we aim to validate the AI with local Thai citizens. We encourage hospitals to validate these solutions using their own data, and to conduct postimplementation studies. The use of AI without these steps could lead to low accuracy, and potentially harmful consequences in the field.” [P14, policy maker, Thailand]

#### Capacity Building for Digital Health Care Transformation

As evidenced in the example quotes from participants P5 and P6 (Table 4), there was an emphasis on enhancing the public’s digital and health literacy to enhance AI readiness in society. Furthermore, equipping health care workers (HCWs) with both soft and technical skills to thrive in a digital-first health care ecosystem was viewed as crucial. These capacity-building efforts tended to be more comprehensive in HICs, where resources allow for continuous professional development, whereas LMICs were at the initial stages of such educational initiatives. This collaborative approach was deemed essential across all country income levels; yet, the specific stakeholders and the focus of collaboration differed, with LMICs often prioritizing public-private partnerships to overcome resource limitations, while HICs focused on refining interagency communication and standardization.

#### Need for Multistakeholder and Interagency Collaboration

Participants across LMICs and HICs emphasized the importance of collaboration across academia, health care, government, and private sectors in facilitating AI implementation and adoption. This insight is particularly illustrated by the example quotes from participants P12, P20, and P21 (Table 4). Participants also advocated for stronger jurisdictional authority, unified standards, leadership development, cross-country learning, and stakeholder engagement to promote effective governance and seamless technology integration.

#### Need for Contextualized Research and Development and Innovation

The scarcity of validation studies was identified as a notable impediment to evidence-based AI implementation, underlining the demand for research that adapts to regional needs to ensure both practical applicability and scientific rigor. It was suggested that localized and validated research and development, including postimplementation studies, be conducted to tailor AI solutions to specific health care contexts. This point is especially evident in the example quote from participant P14 (Table 4). Good research and innovation require good data; without robust and contextually relevant datasets, AI systems risk being poorly adapted or ineffective. This need for contextualized research and development underscored a key difference: while LMICs required research that addressed basic implementation barriers, HICs focused on fine-tuning AI applications to maximize efficiency and effectiveness in already well-resourced systems.

### Data Governance

Data governance emerged as a crucial theme that prioritizes the correct and responsible use of data in health care settings.

#### Need for Effective Data Collection and Management

Participants acknowledged that effective and reliable data collection and management is important for robust AI model development. Several challenges were mentioned, including unclean and unstandardized data, paper-based data, siloed databases, limited real-time data, and the burden of replacing legacy systems. Participants underscored the need for integrating diverse data into a single platform, supported by rigorous validation, expert oversight, and strategies to mitigate algorithmic bias. These challenges were more acute in LMICs, where data infrastructure may be underdeveloped, compared to HICs, where advanced digital systems facilitate more streamlined data management, although complexities in data integration persist.

#### Data Privacy and Security Concerns

Several participants voiced concerns about privacy and security, particularly the challenges associated with anonymizing or deidentifying health data. The example quote from participant P2 (Table 5) supports this interpretation. To enhance secure data collaboration, participants called for stringent data privacy compliance; dedicated resources for data security; and the use of privacy-preserving technologies, such as federated learning [23]. Conversely, others reported that regulatory hurdles posed challenges to AI adoption. In higher-income contexts, the focus often shifted toward sophisticated privacy measures and advanced cybersecurity protocols, reflecting both the abundance of data and the complexity of digital ecosystems.

**Table 5:** Data governance.

Main theme and subthemes			Example quotes
**Data governance**			
	Need for effective data collection and management	“It all comes down to data. In the end, we need data that is of good quality and available in a timely manner for it to be useful. Currently, we don’t have that yet, and we are striving to achieve it. For example, in a hospital, our database may indicate that we have five doctors, but in reality, there may only be three. Furthermore, we don’t have specifics about their specialties. They could be internal medicine doctors, radiologists, or even be on holiday. We don’t have that kind of data yet. Most of it is still paper based or exists in siloed databases that aren’t connected to the registry. So, we don’t even know the real-time resources we have. If we don’t know how many doctors and what specialties are available in hospitals, it’s impossible to utilize AI [artificial intelligence] effectively. Hence, our primary goal is to build a reliable and timely database. From there, we can start training the AI.” [P13, professional from NGO^a^ and multilateral organization, Myanmar] “Most data in hospitals are still unclean, and standard medical terminology has only been adopted in recent years.” [P7, professional from NGO and multilateral organization, Indonesia] “One of the challenges with AI and health systems in many developed regions is that they have to deal with these old legacy systems. In places like the Netherlands, many hospital EHRsb were coded by someone in the 60s using an obscure language that has long been forgotten, and everything has been layered on top of that. Integrating new elements like a new app into such a system is cumbersome. These legacy systems pose a barrier to implementing AI in many high-income regions.” [P11, academic, Singapore]	
	Data privacy and security concerns	“There are still challenges in terms of anonymizing or deidentifying health data so that it can still be utilized without compromising individual privacy.” [P2, academic, Indonesia] “We need to consider security issues, like the potential risk of ransomware or hackers. So, a significant part of the budget would be dedicated to managing security. This also includes the cost of infrastructure, which needs to be sustainable for the government.” [P14, policy maker, Thailand]	
	Need for national server localization	“Having a centralized data repository at a national level would be extremely useful. Currently, AI developers need to build connections with hospitals, hospital by hospital, to gain data for AI development. This approach does not yield large enough datasets to develop robust AI solutions for the Thai population.” [P14, policy maker, Thailand] “I’m also active on the policy side. I’m collaborating with the Thai government on their AI policy, specifically concerning data sharing. We received a grant to identify barriers and facilitators for local data sharing for AI development. Thailand is a small market aiming to expand its local digital health and AI industry, so improving data governance is critical.” [P11, academic, Singapore]	

^a^NGO: nongovernmental organization.

^b^EHR: electronic health record.

#### Need for National Server Localization

Interestingly, some participants pointed to the need for a centralized data repository or national database to improve data access for AI development and ensure data sovereignty and relevance. The example quote from participant P14 (Table 5) sheds light on this issue: in Thailand, AI developers need to establish relationships with individual hospitals to gather data for AI development, but this way of operating does not generate sufficiently large datasets to develop comprehensive AI technologies for the local population. Furthermore, strengthening local data governance was seen as essential for aligning data use with regional regulations and needs. This notion of centralized data governance resonated differently across contexts, with participants from LMICs viewing it as a foundational step toward digital maturity, whereas those from HICs saw it as a means to enhance data interoperability and secure collaborative innovation.

### AI for Health System Transformation

The potential impact of AI on health system transformation was considered monumental, showcasing its considerable opportunities to revolutionize health care and global health both currently and in the future.

#### AI for Population Health Management

The vital role of AI in population health management was highlighted by many participants, particularly in advancing public health monitoring, disease forecasting, and infectious disease control (eg, identifying red or hot spots)—thereby strengthening system preparedness and responsiveness. The quote from participant P12 (Table 6) provides a prime example of how this interpretive narrative was drawn from the data. The potential use of AI in population health policy and intervention development was also thought of as impactful: LMICs focused on leveraging AI to address immediate public health challenges amid resource constraints, whereas HICs were more focused on integrating AI into established health systems for refined policy making.

**Table 6:** Artificial intelligence (AI) for health system transformation.

Main theme and subthemes			Example quotes
**AI for health system transformation**			
	AI for population health management	“AI can analyze data related to public health issues like pandemics, identifying red spots and potential hot spots.” [P12, corporate professional, Indonesia] “When we talk about population health, we can use AI for epidemiology studies, for instance, identifying why a certain community has a higher incidence of diabetes. We can also use AI for outbreak investigations and patient management.” [P17, innovator, Singapore]	
	Improved accessibility to health care services	“AI can definitely bring more efficiency and effectiveness to health care. Especially in remote areas (frontier, lagging, and outermost regions) where access to doctors, health workers, and medicine is challenging. It could be beneficial to establish AI-assisted health facilities, like ATMs [automated teller machines] combined with vending machines. For example, patients can access these facilities at Posyandu [community-based maternal and child health] centers and get necessary health checks through devices that measure various parameters, such as tension, oxygen saturation, and wireless ECG [electrocardiogram]. This AI-driven approach could bring health care closer to people in remote regions, even for those who don’t have smartphones.” [P16, academic, Indonesia]	
	Enhanced health care operations management	“Similarly, in other emerging areas, there are tasks such as organ volume calculation that can be time consuming when done manually. Implementing AI can significantly reduce the time and effort required. For example, instead of spending two hours on manual segmentation, AI could potentially reduce it to just ten minutes. These are the challenges and opportunities that AI presents in the field of medical imaging.” [P20, academic, Thailand] “AI can be leveraged to address manpower shortages. Moving forward, within the next five to ten years, it will have a significant positive impact. We’re also looking to leverage AI to address the gap in human capital. For instance, we’re developing a virtual influenza-like–illness clinic to assist with triaging flu patients. AI can accurately identify the severity of symptoms and direct patients to the appropriate services, thereby freeing up health care workers.” [P28, policy maker, Brunei Darussalam]	
	AI for health systems financing and health care payment	“It could also help to make financial processes more efficient. The current reimbursement process where hospitals send their bills to the government for repayment is cumbersome and inefficient, often resulting in hospitals losing money. If AI can help optimize this process and allocate resources more efficiently, it could be beneficial.” [P23, professional from NGO^a^ and multilateral organization, Thailand] “I’m slightly skeptical about significant changes in life expectancy due to AI, especially in developed countries like Singapore. There, the focus may be more on improving service quality and reducing costs, making it more sustainable in the face of an aging population.” [P11, academic, Singapore]	
	AI for personalized medicine	“People in Singapore are already using AI for diagnoses, among other things.” [P17, innovator, Singapore] “Another effort we are making is to identify potential drug interactions using AI.” [P2, academic, Indonesia] “I think the most basic case, which is quite straightforward, is how it can help patients or anyone in the general population to improve their health. AI can recommend what you should do, what you should eat, or similar actions. If you have a symptom or disease, perhaps it can recommend management strategies for that disease.” [P23, professional from NGO and multilateral organization, Thailand] “So, it’s like mass customization of health care, which is currently impossible because we deliver health from a standard offering. But in the future, we can have these customized delivery and engagement capabilities because of digital health and AI capabilities powering those services.” [P21, investor, Singapore]	

^a^NGO: nongovernmental organization.

#### Improved Accessibility to Health Care Services

Participants indicated that AI has already improved accessibility to health care services and that the trajectory will accelerate. Enhanced remote health care delivery was frequently mentioned as a means by which AI can bridge health care disparities and promote more equitable access to care. To illustrate, a participant from Myanmar shared that telemedicine has become the preferred option for patients because it minimizes both infection risks and barriers regarding physical access to clinics. As another example, a participant commended an AI program sponsored by the Vietnamese government that aims to develop AI systems for remote areas, enhancing access to medical treatment. Participants also suggested that AI could assist in the allocation of physicians and HCWs by identifying both the number needed and the optimal locations for deployment; for instance, an Indonesian participant noted that AI could help address the oversupply of physicians in urban regions and the undersupply in remote areas (refer to the example quote from participant P16 [Table 6]). Furthermore, participants in Singapore believed that in the future, AI tools could support a shift from hospital-based care to community- and home-based models. These examples illustrate how, while LMICs leverage AI to overcome geographic and resource-related challenges, HICs were more inclined toward reimagining care delivery to optimize efficiency and patient-centric models.

#### Enhanced Health Care Operations Management

AI was deemed useful for advancing the management of health care operations, especially in terms of improving efficiency in data analysis and integration across health systems. Advanced performance in triaging and predictive modeling for prognosis and outcomes—enabling mitigation of human error, reduction of readmissions, and prevention of complications—were viewed as possible leaps forward in health care operations management. Improved time efficiency in medical imaging was regarded by participants as a key benefit of AI integration (refer to the example quote from participant P20 [Table 6]). In addition, using AI for better human resource management, such as human resource allocation to alleviate health workforce burden, was regarded as a significant opportunity; for example, participant P28 (Table 6) noted that “AI can be leveraged to address manpower shortages.” This operational enhancement was particularly critical in HICs, where the integration of AI with complex existing systems demanded precise calibration, whereas LMICs still addressed more fundamental operational inefficiencies.

#### AI for Health Systems Financing and Health Care Payment

The advancement of future health systems financing and health care payment emerged as a tangible way in which AI could help transform health systems. Participants shared views on AI’s potential to alleviate state fiscal pressures through targeted service profiling and optimization. Furthermore, AI could have the capability to elevate administrative efficiency in electronic health records (EHRs) and be integrated into personalized medicine workflows—from EHRs to payment processes—resulting in more streamlined financial management in health care. This transformative potential underscored economic disparities: HICs were often in a better position to invest in and implement such integrated systems, while LMICs were still laying the groundwork for basic digital financial management.

#### AI for Personalized Medicine

The current and future effects of AI on advancing personalized medicine was evaluated as profound. Participants discussed the utility of AI for early detection and diagnosis, drug safety and effectiveness, and physician-AI collaboration for clinical decision support. In addition, the role of AI in empowering patient self-management and enabling personalized treatment plans was seen as crucial for promoting individualized care. While the promise of personalized medicine was universally acknowledged, its practical application tended to vary: HICs were more likely to harness the nuanced capabilities of AI in personalized care, whereas LMICs may have initially concentrated on addressing broader diagnostic and treatment challenges.

## Discussion

### Principal Findings and Comparison With Prior Work

To our knowledge, this is one of the largest qualitative studies to examine perspectives on AI adoption in health systems, involving 31 participants from 7 countries across diverse economic levels in SEA. The study identified varying levels of technology acceptance, infrastructure readiness, and governance challenges regarding the development and adoption of AI in LMICs (Myanmar, the Philippines, and Vietnam), UMICs (Indonesia and Thailand), and HICs (Brunei Darussalam and Singapore). Our results offer a comprehensive overview, revealing 5 main themes: AI technology acceptance, digital transformation disparities, technology governance, data governance, and AI for health system transformation.

Generally, participants agreed that high AI technology acceptance is needed for the region to successfully adopt AI in health care. Perceived risks and resistance, including concerns about overreliance, trust, human oversight, and clinical accuracy, may impede acceptance. A survey of Asia-Pacific gastroenterologists indicated general acceptance of AI, with trust levels varying by context (eg, physicians’ experience) [24]. Moreover, the authors concluded that the relationship between perceived risk, acceptance, and trust is complex and requires further research into the factors driving trust and acceptance [24]. However, our study suggests that accessibility and user-friendly systems facilitate AI acceptance. An international review recommended involving user experience researchers throughout the AI development process to enhance usability as well as the impact on human-AI interaction, workflow, and patient outcomes [25].

Systemic barriers to AI adoption arise from significant disparities in digital transformation between LMICs and UMICs. In LMICs, inadequate infrastructure, such as unreliable electricity and internet as well as limited access to digital medical devices, hinders AI adoption and scalability. Internet penetration varies significantly across SEA: Myanmar (44%), the Philippines (53%), and Indonesia (62%) lag behind Vietnam (78%) and Thailand (85%), while Brunei Darussalam (95%) and Singapore (98%) represent high-income digital leaders in the region, highlighting a digital divide impacting AI readiness [26]. A study from Indonesia revealed that 7.5% of primary HCWs working in urban settings have never used the internet, and 15.7% have never operated a computer [27]. Market access issues, including market unpredictability and limited government regulation of private telemedicine sectors, complicate AI scalability in health care. A recent study from Indonesia illustrates a disconnect between central and local government visions for AI policy: while central authorities tend to dominate through top-down regulations and centralized decision-making, local governments often lack the autonomy needed to tailor AI solutions to local contexts [28]. In addition, fiscal constraints, including limited funding, high innovation costs, and low public sector investment, are significant barriers. The Association of Southeast Asian Nations (ASEAN) Secretariat further highlights that, outside of Singapore, SEA countries face infrastructure challenges that impede digital health care advancements [7].

From a technology governance perspective, ethical and regulatory frameworks specific to health care are essential for integrating AI into national health systems [29]. Participants agreed that tailored frameworks are crucial for AI integration, emphasizing legal protections for HCWs; for example, Indonesian physicians have sought legal guidance on medical liability regarding the use of AI for clinical decision-making. Participants also emphasized capacity building, multistakeholder collaboration, and the importance of localized research and development to ensure that AI tools are accurate and contextually appropriate. In addition, AI technologies require continuous monitoring after deployment to ensure accuracy, appropriate use, and clinical effectiveness, as well as to reduce bias. Ethical and regulatory frameworks should include monitoring and audits to minimize patient harm and medical liability.

Participants from HICs and UMICs emphasized the need for effective data management and local data residency on national servers to ensure personal data privacy and security. This aligns with the ASEAN Secretariat’s recommendation for SEA countries to enhance standards and interoperability in digital health services [7]. Globally, data interoperability is widely discussed in the contexts of health services management, patient data, diagnostics, and clinical decision-making, with important implications for patient safety and care quality [30]. Standardized ethical practices are supported by the Canada-based Centre for Advancing Responsible & Ethical Artificial Intelligence [31]; and the US-based Coalition for Health AI, through its Assurance Standards Guide [32]. In SEA, Duke-NUS Medical School in Singapore has introduced the Transparent Reporting of Ethics for Generative AI checklist, a standardized ethics checklist for generative AI research in health care [33].

Participants from all country income groups acknowledged AI’s potential to transform health systems by advancing population health management; improving accessibility; enhancing operations; and supporting financing, payment systems, and personalized medicine. AI aids in population health management, including infectious disease control, public health monitoring, and disease forecasting. During the COVID-19 pandemic, unprecedented challenges to global public health prompted the adoption of AI technologies across various sectors, including health care, in SEA; for example, the Singapore government used AI-enabled digital contact tracing to help mitigate SARS-CoV-2 transmission, reducing morbidity and mortality [34].

Some participants noted that AI could improve accessibility to health care services through AI-enabled telemedicine; for example, an Indonesian telemedicine platform uses an AI chatbot to prescreen patients, providing prediagnosis recommendations to physicians and streamlining patient consultations in remote areas [35]. Participants also shared that AI may optimize health systems financing by streamlining EHRs, enhancing service profiling, and integrating personalized medicine workflows. A study from Singapore described the use of an AI prediction model and data integration to identify patients at high risk of multiple hospital readmissions, facilitating targeted interventions aimed at improving patient outcomes and potentially reducing health care costs [36].

Participants suggested that, to alleviate workforce burdens and improve patient outcomes, AI could be used to enhance health care operations by advancing data integration, predictive modeling, and medical imaging; reducing human error; optimizing triaging; and improving human resource management. In Singapore, studies show that AI supports health care operations by streamlining medical records, optimizing resources, and reducing operations costs [36]. AI can profoundly impact personalized medicine by supporting early diagnosis, drug safety, clinical decision-making, and patient self-management. In Thailand, personalized medicine is currently applied in oncology, where physicians use supercomputer analytics to formulate optimal cancer treatment plans [37]. Despite AI’s transformative role across health care ecosystems in SEA, adoption remains uneven, necessitating stakeholder collaboration and partnership for optimal integration across the region [8].

### Strengths

This study includes several strengths. To our knowledge, it is one of the largest qualitative studies exploring the unique perspectives of participants from 7 countries across different economic and AI-readiness levels in SEA. Engaging diverse participants involved in AI implementation in SEA’s health care sector contributes significantly to the growing field of AI in this underresearched region. It also provides a foundation for future research to adopt a multistakeholder lens when assessing AI adoption globally.

### Limitations

This study has a number of limitations. First, due to the qualitative nature of our study, the perspectives gathered cannot be generalized to the broader population. Nevertheless, we included a wide range of participants from HICs, UMICs, and LMICs across 7 (70%) out of 10 SEA countries.

Second, the disproportionate number of participants from Indonesia (12/31, 39%)—a country with a population of approximately 281 million, accounting for >40% of the total ASEAN population of 661 million—may have also skewed the findings toward Indonesian-specific challenges and opportunities. Conversely, the limited representation from the Philippines (1/31, 3%; population of 115 million) and Brunei Darussalam (1/31, 3%; population of 0.4 million), along with the absence of participants from Malaysia, Laos, and Cambodia, may have constrained the range of insights into the diverse health care systems, levels of infrastructure readiness, and policy environments across the region. This imbalance could affect the interpretation of results when drawing conclusions about regional trends because the perspectives of larger or more digitally advanced countries may overshadow those of smaller or underrepresented nations, limiting the identification of subregional patterns such as those in the Mekong or Borneo regions.

Finally, 84% (26/31) of the participants were male, which may limit perspectives related to gender-specific experiences and viewpoints regarding health AI adoption. Gender dynamics influence both health care delivery and technology use, and male-dominated views may overlook issues such as gender bias in AI, disparities in women’s access to digital tools, and variations in digital literacy by gender. As AI systems operate within social contexts shaped by gender norms, this imbalance may constrain the understanding of diverse user needs and risk reinforcing existing inequalities. Future studies should ensure greater gender diversity among informants, especially in leadership, clinical, and technical roles, to develop more equitable and effective AI health solutions.

### Conclusions

Our study provides new insights into the factors affecting the adoption of AI in health systems across SEA. While certain views were consistent across different economic levels, others were unique to specific groups, highlighting the importance of locally contextualizing AI research and innovation. Interviewees from various professional groups and countries identified common issues but often focused on aspects that they deemed most relevant in their local context.

Transforming health systems with AI requires significant investment in digital infrastructure because uneven development limits scalability and integration. Effective AI implementation requires robust technology and data governance frameworks to ensure ethical development, data privacy, and reliable integration. Coordinated action among policy makers, health care providers, technology developers, government agencies, and the private sector is essential to align strategies and enhance the resilience of health systems across SEA. Coordinated efforts between governments and private sector organizations are often motivated by shared challenges and the leveraging of resources. Future research should focus on value-based AI development shared across the region. Moreover, socioeconomic differences between nations offer opportunities for cross-border partnerships; learning from each other’s experiences; and adapting policies to enhance skills development, cost savings, and shared access to innovative technology and deidentified data—resources that may otherwise be unattainable within a single country’s socioeconomic situation.

## Appendix

Multimedia Appendix 1 COREQ 32-item checklist.

Multimedia Appendix 2 Interview guide.

Multimedia Appendix 3 Extended data collection and analysis.

## Abbreviations

AI: artificial intelligence
ASEAN: Association of Southeast Asian Nations
COREQ: Consolidated Criteria for Reporting Qualitative Research
EHR: electronic health record
HCW: health care worker
HIC: high-income country
LMIC: lower–middle-income country
PHC: primary health care
SEA: Southeast Asia
UHC: universal health coverage
UMIC: upper–middle-income country

## Data Availability

The datasets generated or analyzed during this study are available from the corresponding author on reasonable request.

## References

[1] Borges do Nascimento IJ, Abdulazeem H, Vasanthan L, Martinez E, Zucoloto M, Østengaard L, Azzopardi-Muscat N, Zapata T, Novillo-Ortiz D (2023). The global effect of digital health technologies on health workers’ competencies and health workplace: an umbrella review of systematic reviews and lexical-based and sentence-based meta-analysis. Lancet Digit Health.

[2] Ciecierski-Holmes T, Singh R, Axt M, Brenner S, Barteit S (2022). Artificial intelligence for strengthening healthcare systems in low- and middle-income countries: a systematic scoping review. NPJ Digit Med.

[3] Bergeri I, Whelan MG, Ware H, Subissi L, Nardone A, Lewis HC, Li Z, Ma X, Valenciano M`, Cheng B`, Al Ariqi L, Rashidian A, Okeibunor J, Azim T, Wijesinghe P, Le LV, Vaughan A, Pebody R, Vicari A, Yan T, Yanes-Lane M, Cao C, Clifton DA, Cheng MP, Papenburg J, Buckeridge D, Bobrovitz N, Arora RK, Van Kerkhove MD (2022). Global SARS-CoV-2 seroprevalence from January 2020 to April 2022: a systematic review and meta-analysis of standardized population-based studies. PLoS Med.

[4] Schwalbe N, Wahl B (2020). Artificial intelligence and the future of global health. The Lancet.

[5] Tan TF, Thirunavukarasu AJ, Jin L, Lim J, Poh S, Teo ZL, Ang M, Chan RV, Ong J, Turner A, Karlström J, Wong TY, Stern J, Ting DS (2023). Artificial intelligence and digital health in global eye health: opportunities and challenges. Lancet Glob Health.

[6] Kumar Y, Koul A, Singla R, Ijaz MF (2023). Artificial intelligence in disease diagnosis: a systematic literature review, synthesizing framework and future research agenda. J Ambient Intell Humaniz Comput.

[7] Croissant A, Lorenz P (2017). Comparative Politics of Southeast Asia: An Introduction to Governments and Political Regimes.

[8] Diana A (2023). Transforming the digital health landscape in ASEAN. ASEAN Socio-Cultural Community Policy Brief.

[9] (2021). Global strategy on digital health 2020-2025. World Health Organization.

[10] McLennan S, Fiske A, Celi LA (2024). Building a house without foundations? A 24-country qualitative interview study on artificial intelligence in intensive care medicine. BMJ Health Care Inform.

[11] Dhillon I, Jhalani M, Thamarangsi T, Siyam A, Singh PK (2023). Advancing universal health coverage in the WHO South-East Asia region with a focus on human resources for health. Lancet Reg Health Southeast Asia.

[12] Weinert L, Müller J, Svensson L, Heinze O (2022). Perspective of information technology decision makers on factors influencing adoption and implementation of artificial intelligence technologies in 40 German hospitals: descriptive analysis. JMIR Med Inform.

[13] Hummelsberger P, Koch TK, Rauh S, Dorn J, Lermer E, Raue M, Hudecek MF, Schicho A, Colak E, Ghassemi M, Gaube S (2023). Insights on the current state and future outlook of AI in health care: expert interview study. JMIR AI.

[14] Secinaro S, Calandra D, Secinaro A, Muthurangu V, Biancone P (2021). The role of artificial intelligence in healthcare: a structured literature review. BMC Med Inform Decis Mak.

[15] Tran BX, Vu GT, Ha GH, Vuong QH, Ho MT, Vuong TT, La VP, Ho MT, Nghiem KC, Nguyen HL, Latkin CA, Tam WW, Cheung NM, Nguyen HK, Ho CS, Ho RC (2019). Global evolution of research in artificial intelligence in health and medicine: a bibliometric study. J Clin Med.

[16] Chongsuvivatwong V, Phua KH, Yap MT, Pocock NS, Hashim JH, Chhem R, Wilopo SA, Lopez AD (2011). Health and health-care systems in southeast Asia: diversity and transitions. The Lancet.

[17] Braun V, Clarke V (2006). Using thematic analysis in psychology. Qual Res Psychol.

[18] Long Q, Jiang H (2023). Qualitative research in health: value and visibility. Lancet Reg Health West Pac.

[19] Tong A, Sainsbury P, Craig J (2007). Consolidated criteria for reporting qualitative research (COREQ): a 32-item checklist for interviews and focus groups. Int J Qual Health Care.

[20] Marshall MN (1996). Sampling for qualitative research. Fam Pract.

[21] Otter.ai.

[22] Metreau E, Young KE, Eapen SG (2024). World Bank country classifications by income level for 2024-2025. World Bank Blogs.

[23] Li S, Liu P, Nascimento GG, Wang X, Leite FR, Chakraborty B, Hong C, Ning Y, Xie F, Teo ZL, Ting DS, Haddadi H, Ong ME, Peres MA, Liu N (2023). Federated and distributed learning applications for electronic health records and structured medical data: a scoping review. J Am Med Inform Assoc.

[24] Goh W, Chia K, Cheung M, Kee K, Lwin M, Schulz P, Chen M, Wu K, Ng SS, Lui R, Ang TL, Yeoh KG, Chiu HM, Wu DC, Sung JJ (2024). Risk perception, acceptance, and trust of using AI in gastroenterology practice in the Asia-Pacific region: web-based survey study. JMIR AI.

[25] Asan O, Choudhury A (2021). Research trends in artificial intelligence applications in human factors health care: mapping review. JMIR Hum Factors.

[26] Internet adoption in Southeast Asia as of February 2025, by select country. Statista.

[27] (2019). Digital health literacy and internet behavior of primary health care staff in Indonesia, are they ready adopting eHealth?. Asian Health Literacy Association.

[28] Wadipalapa RP, Katharina R, Nainggolan PP, Aminah S, Apriani T, Ma’rifah D, Anisah AL (2024). An ambitious artificial intelligence policy in a decentralised governance system: evidence from Indonesia. J Curr Southeast Asian Aff.

[29] Ong JC, Seng BJ, Law JZ, Low LL, Kwa AL, Giacomini KM, Ting DS (2024). Artificial intelligence, ChatGPT, and other large language models for social determinants of health: current state and future directions. Cell Rep Med.

[30] Li E, Clarke J, Ashrafian H, Darzi A, Neves AL (2022). The impact of electronic health record interoperability on safety and quality of care in high-income countries: systematic review. J Med Internet Res.

[31] Centre for Advancing Responsible and Ethical Artificial Intelligence (CARE-AI) homepage. Centre for Advancing Responsible and Ethical Artificial Intelligence (CARE-AI).

[32] Hill B (2024). CHAI releases draft responsible health AI framework for public comment. CHAI - Coalition for Health AI.

[33] Ning Y, Teixayavong S, Shang Y, Savulescu J, Nagaraj V, Miao D, Mertens M, Ting DS, Ong JC, Liu M, Cao J, Dunn M, Vaughan R, Ong ME, Sung JJ, Topol EJ, Liu N (2024). Generative artificial intelligence and ethical considerations in health care: a scoping review and ethics checklist. Lancet Digit Health.

[34] Farhat F, Sohail SS, Alam MT, Ubaid S, Ashhad M, Madsen DØ, Shakil (2023). COVID-19 and beyond: leveraging artificial intelligence for enhanced outbreak control. Front Artif Intell.

[35] Nur Aisyah D, Lokopessy AF, Naman M, Diva H, Manikam L, Adisasmito W, Kozlakidis Z (2023). The use of digital technology for COVID-19 detection and response management in Indonesia: mixed methods study. Interact J Med Res.

[36] Abisheganaden J, Lee KH, Low LL, Shum E, Goh HL, Ang CG, Ta AW, Miller SM (2023). Lessons learned from the hospital to home community care program in Singapore and the supporting AI multiple readmissions prediction model. Health Care Sci.

[37] Suwanvecho S, Suwanrusme H, Jirakulaporn T, Issarachai S, Taechakraichana N, Lungchukiet P, Decha W, Boonpakdee W, Thanakarn N, Wongrattananon P, Preininger AM, Solomon M, Wang S, Hekmat R, Dankwa-Mullan I, Shortliffe E, Patel VL, Arriaga Y, Jackson GP, Kiatikajornthada N (2021). Comparison of an oncology clinical decision-support system's recommendations with actual treatment decisions. J Am Med Inform Assoc.

[38] ChatGPT.

