# Supplementary material for: Insights Into the Current and Future State of AI Adoption Within Health Systems in Southeast Asia: Cross-Sectional Qualitative Study
Source: J Med Internet Res. 2025 Jun 16;27:e71591. doi: 10.2196/71591 (PMC12209719; doi:10.2196/71591)
Supplement: Multimedia Appendix 2 [file jmir_v27i1e71591_app2.docx]

**Multimedia appendix 2: Interview guide**

1. Would you please share a story about how AI is already making a difference in your own life? This does not necessarily have to be related to health.

2. How do you imagine AI can be used to improve health outcomes, or protect the health of populations, and strengthen healthcare systems? Could you provide any specific examples of how this is already happening?

3. What do you think are the potential challenges that will be faced in creating and using AI for health in Singapore (only to be asked to participants in Singapore) (or) in settings with limited resources? Please share specific examples.

4. Do you have any concerns about how AI will be developed or used in your line of work? Can you share an example from real life?

5. What do you think will be the impacts of AI on healthcare in country X (country of relevance depending on location and/or operations of individual) (or) Southeast Asia over the next 5 to 10 years? What actions should we take now to ensure that AI benefits people over these coming years?

6. What is one of the most burning questions in your area of work that you would want a highly capable AI system to be able to answer? What actions should be taken now to ensure that AI benefits people during this period?
