# Supplementary material for: Insights Into the Current and Future State of AI Adoption Within Health Systems in Southeast Asia: Cross-Sectional Qualitative Study
Source: J Med Internet Res. 2025 Jun 16;27:e71591. doi: 10.2196/71591 (PMC12209719; doi:10.2196/71591)
Supplement: Multimedia Appendix 3 [file jmir_v27i1e71591_app3.docx]

**Multimedia appendix 3: Extended data collection and analysis**

**Extended data collection: Reflexivity**

MFW., a male researcher (MD) in global health innovation and an Indonesian national, conducted twenty-seven interviews. JK., a Singapore-based associate professor (MD) in global health innovation and a Swedish national, conducted the remaining four interviews. Most interviews were conducted in English. Eight key informants coming from Indonesia were interviewed in Bahasa Indonesia to accommodate interviewees’ limited proficiency in professional English. There was no hierarchical relationship between the interviewers and the study participants.

**Extended data analysis**

The following table presents the six phases of thematic analysis^1^ along with a description of how each phase was applied in this study. Although the analysis procedure is described here with distinct steps, it is a cyclical, iterative process that requires several refinements to reach the final thematic tables.

| **Phases of thematic analysis** | **Description of phase applied to the study** |
| --- | --- |
| Phase 1: Data familiarisation and writing  familiarisation notes | The interview audio-recordings were transcribed using Otter.ai.^2^ MFW checked all transcripts for accuracy and translated the Indonesian transcripts into English. |
| Phase 2: Systematic data coding | To enhance intercoder reliability, MFW, EL and A.P coded the data independently and then convened once all transcripts had been coded to check for coherence. Conversations among the investigators (MFW, EL and AP) continued until any minor coding differences were resolved and consensus was achieved. |
| Phase 3: Generating initial themes from  coded data and collated data | Each transcript’s codes with representative quotes were transferred into a Microsoft Excel spreadsheet by A.P. Related codes were then categorised into initial sub-themes and main themes. |
| Phase 4: Developing and reviewing themes | To strengthen intracoder reliability, AP re-coded a sample of the data and compared the initial and subsequent coding to ensure the codes consistently reflect the intended sub-themes and main themes. A thematic table consisting of the main themes, sub-themes and codes was subsequently  developed by AP. |
| Phase 5: Refining, defining and naming  themes | To establish names and meanings for the  themes that best capture the essence of the data, the thematic table was refined by AP, further reviewed by MFW, and conclusively finalised by AP. |
| Phase 6: Writing the report | AP extracted in-depth and representative participant quotes with associated sub-themes and main themes from the refined Microsoft Excel spreadsheet and collated them into the final thematic tables and results. The results were then compared and contrasted to the study aims and existing literature. |

**Ethical considerations**

This study received institutional review board exemption under NUS-IRB Reference Code: NUS-IRB-2023-562 from the National University of Singapore. Verbal consent was obtained from all participants before the interviews and audio recordings commenced.

**References**

1. Braun, V., & Clarke, V. (2006). Using thematic analysis in psychology. *Qualitative Research in Psychology*, 3(2), 77–101. <https://doi.org/10.1191/1478088706qp063oa>

2. Otter.ai. *AI Meeting Note Taker & Real-time AI Transcription*. Otter.ai. <https://otter.ai/>. Published 2023; Accessed 2023 Aug.
